# *Lactobacillus acidophilus* Supplementation Exerts a Synergistic Effect on Tacrolimus Efficacy by Modulating Th17/Treg Balance in Lupus-Prone Mice *via* the SIGNR3 Pathway

**DOI:** 10.3389/fimmu.2021.696074

**Published:** 2021-12-10

**Authors:** Da Som Kim, Youngjae Park, Jeong-Won Choi, Sung-Hwan Park, Mi-La Cho, Seung-Ki Kwok

**Affiliations:** ^1^ The Rheumatism Research Center, Catholic Research Institute of Medical Science, College of Medicine, The Catholic University of Korea, Seoul, South Korea; ^2^ Laboratory of Translational ImmunoMedicine, Catholic Research Institute of Medical Science, College of Medicine, The Catholic University of Korea, Seoul, South Korea; ^3^ Department of Biomedicine & Health Sciences, College of Medicine, The Catholic University of Korea, Seoul, South Korea; ^4^ Division of Rheumatology, Department of Internal Medicine, Seoul St. Mary’s Hospital, College of Medicine, The Catholic University of Korea, Seoul, South Korea; ^5^ Department of Medical Lifescience, College of Medicine, The Catholic University of Korea, Seoul, South Korea

**Keywords:** systemic lupus erythematosus, gut microbiome, *Tacrolimus*, *Lactobacillus acidophilus*, T helper 17 cell, regulatory T cell

## Abstract

**Objective:**

Tacrolimus (Tac) is an immunosuppressant used in the treatment of systemic lupus erythematosus (SLE); however, it induces T cell subset imbalances by reducing regulatory T (Treg) cells. *Lactobacillus acidophilus* (LA) is reported to have therapeutic efficacy in immune-mediated diseases *via* T cell regulation.

**Methods:**

This study investigated whether a combination therapy of LA and Tac improves the therapeutic efficacy of Tac by modulating T cell subset populations in an animal model of SLE. Eight-week-old MRL/*lpr* mice were orally administered with 5 mg/kg of Tac and/or 50 mg/kg of LA daily for 8 weeks. Cecal microbiota compositions, serum autoantibodies levels, the degree of proteinuria, histological changes in the kidney, and populations of various T cell subsets in the spleen were analyzed.

**Results:**

Mice presented with significant gut dysbiosis, which were subsequently recovered by the combination treatment of Tac and LA. Double negative T cells in the peripheral blood and spleens of MRL/*lpr* mice were significantly decreased by the combination therapy. The combination treatment reduced serum levels of anti-dsDNA antibodies and Immunoglobulin G2a, and renal pathology scores were also markedly alleviated. The combination therapy induced Treg cells and decreased T helper 17 (Th17) cells both *in vitro* and *in vivo*. *In vitro* treatment with LA induced the production of indoleamine-2,3-dioxygenase, programmed death-ligand 1, and interleukin-10 *via* the specific intracellular adhesion molecule-3 grabbing non-integrin homolog-related 3 receptor signals.

**Conclusion:**

The present findings indicate that LA augments the therapeutic effect of Tac and modulates Th17/Treg balance in a murine model of SLE.

## Introduction

Systemic lupus erythematosus (SLE) is a chronic autoimmune disease characterized by the presence of tissue-binding autoantibodies and the formation of immune complexes (1). SLE can affect multiple organ systems concurrently and have fatal consequences due to damage to organs such as the kidneys or the central nervous system (1). Its pathophysiology likely involves the innate and adaptive immune systems, as well as environmental and genetic factors; however, its clinical complexity and heterogeneity remain a challenge (2).

Currently, there is no cure for SLE; most therapeutic modalities focus on nonspecific immune suppression (1, 3). Tacrolimus (Tac), also known as FK506, is one of the most widely used immunosuppressants in organ transplant recipients and patients with autoimmune diseases, including those with lupus nephritis (LN); it inhibits calcineurin, thereby suppressing T cell development and proliferation (4, 5). However, Tac also represses the production of interleukin (IL)-2, which is essential for regulatory T (Treg) cell function (6–8). Reduction in Treg cell production can cause an imbalance in T helper 17 (Th17) and Treg cell numbers, leading to dysfunctions in immune regulation; in fact, an imbalanced Th17/Treg ratio has been suggested as a pathognomonic immune alteration of SLE (9). For these reasons, the therapeutic role and efficacy of Tac can be limited in SLE.

Changes in gut microbiota are observed in various autoimmune diseases (10). Bacterial dysbiosis is seen in SLE patients and, to some extent, in murine models of lupus (11–13). Furthermore, the dysbiosis is not only detected in the intestinal microbiota, but also in the oral mucosa and skin of lupus-affected subjects (14, 15). Therefore, bacterial dysbiosis may be related to the clinical status, or even the dietary metabolism, of lupus patients (16, 17). Whether changes in the cecal bacterial composition of lupus patients are merely coincidental, or are genuine etiological factors of the disease, are unknown; however, probiotic implantation as a novel therapeutic modality in SLE shows some promises (18). More specifically, the administration of probiotics has been demonstrated to improve lupus-related clinical features and reduce inflammatory cytokines in lupus-prone and lupus-induced mouse models (18, 19). Probiotic treatment also affected the T cell subset population, represented by a skewing of the Th17/Treg ratio towards an immune-regulatory phenotype. Moreover, this effect was not limited to the gut mucosa or its associated lymphoid tissues, but instead was systemic (19).

Against this background, probiotic supplementation is hypothesized to augment the therapeutic efficacy of Tac in SLE by limiting its destabilizing effects on the Th17/Treg ratio. Considering the past studies demonstrating decreased proportions of *Lactobacillus* in lupus-prone mice, we chose one of the species included in this genus (11–13). The preclinical data using animal models for osteoarthritis and colitis suggest *Lactobacillus acidophilus* (LA) as a candidate for the immune modulators (20–23). Furthermore, the specific intracellular adhesion molecule-3 grabbing non-integrin homolog-related 3 (SIGNR3), a specific C-type lectin receptor of immune cells, is supposed to act as a key mediator to link between LA and immune cells of the host according to the previous literature (20). To investigate whether Tac and probiotic supplementation is more effective for treating lupus when administered in combination rather than separately, the gut microbiota were assessed before and after the development of a lupus-like phenotype in an animal model of SLE. Then, Tac with and without probiotics was administered, and efficacy was compared by various measures.

## Materials and Methods

### Animals

MRL/MpJ-*Fas^lpr^* (MRL/*lpr*) mice were purchased from SLC (Shizuoka, Japan). They were housed in groups of five in polycarbonate cages in a specific-pathogen-free environment. The mice had access to standard mouse chow (Ralston Purina, St. Louis, MO, USA) and water *ad libitum*. Eight-week-old MRL/*lpr* mice were orally administered 5 mg/kg Tac (MedChemExpress, Monmouth Junction, NJ, USA) and/or 50 mg/kg LA (CNS Pharm Korea, Ltd., Jincheon, Korea), daily for 8 weeks. The LA were heat-killed at 80°C for 30 min prior to administration. All experimental procedures were approved by the Animal Research Ethics Committee of the Catholic University of Korea (approval number: 2020-0151-04).

### Cecal DNA Extraction, Polymerase Chain Reaction Amplification and Sequencing

Total DNA was extracted using the Maxwell RSC PureFood GMO and Authentication Kit (Promega, Madison, WI, USA), in accordance with the manufacturer’s instruction. Polymerase chain reaction (PCR) amplification was performed using fusion primers targeting from V3 to V4 regions of the 16S rRNA gene with the extracted DNA. For bacterial amplification, fusion primers of 341F (5’-AATGATACGGCGACCACCGAGATCTACAC-XXXXXXXX-TCGTCGGCAGCGTC-AGATGTGTATAAGAGACAG-CCTACGGGNGGCWGCAG-3’) and 805R (5’- CAAGCAGAAGACGGCATACGAGAT-XXXXXXXX-GTCTCGTGGGCTCGG-AGATGTGTATAAGAGACAG-GACTACHVGGGTATCTAATCC-3’). The Fusion primers were constructed in the following order which is P5 (P7) graft binding, i5 (i7) index, Nextera consensus, Sequencing adaptor, and Target region sequence. The amplifications were carried out under the following conditions: initial denaturation at 95°C for 3 min, followed by 25 cycles of denaturation at 95°C for 30 sec, primer annealing at 55°C for 30 sec, and extension at 72°C for 30 sec, with a final elongation at 72°C for 5 min. The PCR product was confirmed by using 1% agarose gel electrophoresis and visualized under a Gel Doc system (Bio-Rad, Hercules, CA, USA). The amplified products were purified with the CleanPCR (CleanNA, Waddinxveen, Netherlands). Equal concentrations of purified products were pooled together and removed short fragments (non-target products) with CleanPCR (CleanNA). The quality and product size were assessed on a Bioanalyzer 2100 (Agilent, Palo Alto, CA, USA) using a DNA 7500 chip. Mixed amplicons were pooled and the sequencing was carried out at ChunLab (Seoul, Korea), with Illumina MiSeq Sequencing system (Illumina, San Diego, CA, USA) according to the manufacturer’s instructions.

### Cecal Microbiome Data Analysis Pipeline

Processing raw reads started with quality check and filtering of low quality (< Q25) reads by Trimmomatic version 0.32 (24). After QC pass, paired-end sequence data were merged together using fastq_mergepairs command of VSEARCH version 2.13.4 (25) with default parameters. Primers were then trimmed with the alignment algorithm of Myers & Miller (26) at a similarity cut off of 0.8. Non-specific amplicons that do not encode 16S rRNA were detected by nhmmer (27) in HMMER software package version 3.2.1 with hmm profiles. Unique reads were extracted and redundant reads were clustered with the unique reads by derep_fulllength command of VSEARCH (25). The EzBioCloud 16S rRNA database (28) was used for taxonomic assignment using usearch_global command of VSEARCH (25) followed by more precise pairwise alignment (26). Chimeric reads were filtered on reads with < 97% similarity by reference based chimeric detection using UCHIME algorithm (29) and the non-chimeric 16S rRNA database from EzBioCloud. After chimeric filtering, reads that are not identified to the species level (with < 97% similarity) in the EzBioCloud database were compiled and cluster_fast command (25) was used to perform *de novo* clustering to generate additional operational taxonomic units (OTUs). Finally, OTUs with single reads (singletons) were omitted from further analysis. The secondary analysis which includes diversity calculation and biomarker discovery was conducted in EzBioCloud 16S-based MTP, which is a ChunLab bioinformatics cloud platform.

### Flow Cytometry

Splenocytes and peripheral blood were immunostained with surface eFluor780-conjugated fixable viability dye (eBioscience, San Diego, CA, USA), Pacific Blue-conjugated anti-CD90.2 (BioLegend, San Diego, CA, USA), peridinin-chlorophyll-protein-cyanine5.5-conjugated anti-CD4 (eBioscience), phycoerythrin (PE)-conjugated anti-CD8 (BioLegend), and allophycocyanin (APC)-conjugated anti-CD2 (BioLegend). After fixation and permeabilization, cells were stained with fluorescein isothiocyanate (FITC)-conjugated IL-17 (eBioscience) and PE-conjugated forkhead box P3 (Foxp3; eBioscience). For intracellular staining, cells were stimulated with 25 ng/ml phorbol 12-myristate 13-acetate and 250 ng/mL ionomycin (Sigma, St. Louis, MO, USA) for 4 h in the presence of GolgiStop (BD Biosciences, San Diego, CA, USA). The data were analysed using FlowJo software (Tree Star, Ashland, OR, USA).

### Histological Analysis

Histological analyses were performed to quantify spleen and kidney inflammation. Kidney tissues were fixed in 4% paraformaldehyde, embedded in paraffin, and sectioned. Spleen tissue cryosections were fixed in methanol-acetone. Kidney and spleen sections were stained with hematoxylin and eosin, examined under a photomicroscope (Olympus, Tokyo, Japan), and scored (30).

### Immunohistochemistry

Immunohistochemistry was performed using a Vectastain ABC Kit (Vector Laboratories, Burlingame, CA, USA). Briefly, tissue sections were incubated overnight at 4°C with a primary antibody against SIGNR3 (R&D Systems, Minneapolis, MN, USA), followed by a biotinylated secondary antibody, and then reacted with a streptavidin-peroxidase complex for 1 h. 3,3’-Diaminobenzidine (Dako, Carpinteria, CA, USA) was added as a chromogen, and the samples were visualized using a microscope (Olympus).

### Confocal Microscopy

Tissue cryosections (7 µm-thick) were fixed in methanol-acetone and stained with FITC-conjugated anti-CD4, APC-conjugated anti-CD25, PE-conjugated anti-IL-17, and -Foxp3 (eBioscience). After incubation at 4°C overnight, the stained sections were analysed using a Zeiss LSM 510 Meta microscope (Carl Zeiss, Oberkochen, Germany) at ×200 magnification.

### Enzyme-Linked Immunosorbent Assay

Blood was collected from the orbital sinus, and serum samples were stored at -20°C until use. Serum levels of anti-double stranded DNA (dsDNA) antibodies were measured using poly-L-lysine, dsDNA-cellulose (Sigma), and mouse immunoglobulin G (IgG) detection antibody (Bethyl Laboratories, Montgomery, TX, USA). IgG2a levels were measured using enzyme-linked immunosorbent assay (ELISA) kits (Bethyl Laboratories). The levels of IL-10 and IL-17 in the cultured supernatants from MRL/*lpr* splenocytes were measured using sandwich ELISA (R&D Systems). Absorbances were determined using an ELISA microplate reader (Molecular Devices, Sunnyvale, CA, USA).

### Urine Albumin and Creatinine Assays

Spot urine samples were collected using aseptic microtubes. Urine albumin and creatinine concentrations were measured using a mouse albumin ELISA assay (Bethyl Laboratories) and a creatinine assay (R&D systems), respectively, according to the manufacturer’s instructions.

### Real-Time PCR

mRNA was extracted using TRI Reagent (Molecular Research Center, Cincinnati, OH, USA) as per the manufacturer’s instructions. Complementary DNA was synthesized using a Super Script reverse transcription system (TaKaRa, Shiga, Japan). A Light-Cycler 2.0 Instrument (software version 4.0; Roche Diagnostics, Indianapolis, IN, USA) was used for the PCR amplification. All reactions were performed using the LightCycler FastStart DNA Master SYBR Green I Mix (TaKaRa) following the manufacturer’s instructions. The following primers were used: SIGNR3, 5’-TCA-AGA-GTT-TGG-CAG-AGT-ATA-CG-3’ (sense) and 5’-TTG-TTC-TGA-ACC-TCT-GAG-CTG-3’ (antisense); indoleamine-2,3-dioxygenase 1 (IDO1), 5’-GAC-GGA-CTG-AGA-GGA-CAC-AG-3’ (sense) and 5’-GGC-AGC-ACC-TTT-CGA-ACA-TC-3’ (antisense); programmed death-ligand 1 (PD-L1), 5’-AAA-GTC-AAT-GCC-CCA-TAC-CG-3’ (sense) and 5’-TTC-TCT-TCC-CAC-TCA-CGG-GT-3’ (antisense); IL-10, 5’-GGC-CCA-GAA-ATC-AAG-GAG-CA-3’ (sense) and 5’-AGA-AAT-CGA-TGA-CAG-CGC-CT-3’ (antisense); β-actin, 5’-GAA-ATC-GTG-CGT-GAC-ATC-AAA-G-3’ (sense) and 5’-TGT-AGT-TTC-ATG-GAT-GCC-ACA-G-3’ (antisense). All mRNA levels were normalized to β-actin.

### Small Interfering RNA Transfection

Small interfering RNA (siRNA) for SIGNR3 was purchased from Cosmo Genetech (Seoul, Korea). Before transfection, murine non-T cells were cultured with LPS (100 ng/mL; Sigma) from *Escherichia coli* O111:B4. The next day, the cells were transfected using the Amaxa 4D-nucleofector X unit with a primary cell kit, as per the manufacturer’s recommendations (Lonza, Cologne, Germany).

### Peripheral Blood Mononuclear Cell Isolation and Stimulation

Peripheral blood mononuclear cells (PBMCs) of healthy volunteers (n = 5) and SLE patients (n = 9) were prepared from heparinized blood using a standard Ficoll-Paque density gradient centrifugation (GE Healthcare Biosciences, Uppsala, Sweden). Cells were cultured in RPMI-1640 medium (Gibco BRL, Carlsbad, CA, USA) and stimulated with anti-CD3 (0.5 µg/mL) for 3 days. All procedures were approved by the ethics committee of Seoul St. Mary’s Hospital (Seoul, Korea).

### Statistics

Statistical analyses were performed using GraphPad Prism (version 5.0; GraphPad Software, San Diego, CA, USA). Differences between groups were evaluated using t-tests (two-tailed) for two groups and one-way analysis of variance for three or more groups. *P* < 0.05 was considered statistically significant.

## Results

### Gut Dysbiosis of Lupus-Prone Mice

**Figure 1:**
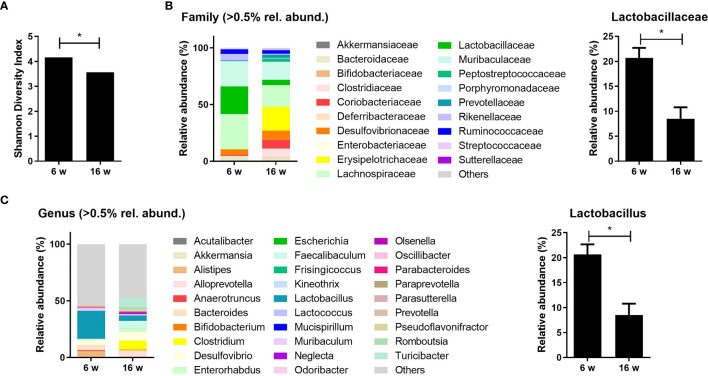
Lupus-prone mice exhibit gut dysbiosis with a reduction in *Lactobacillus* species. **(A)** Shannon diversity indices of the cecal microbiota from MRL/*lpr* mice at 6 and 16 weeks old (*n* = 4). **(B, C)** The relative abundance of cecal microbiota at the family and genus levels in MRL/*lpr* mice (*n* = 3) at 6 and 16 weeks old. The relative abundance of *Lactobacillaceae* **(B)** and *Lactobacillus* **(C)**. All experiments were performed at least three times in triplicate. Data are shown as mean ± SEM. *p < 0.05.

MRL/*lpr* mice were used as a murine model for lupus in this study because they show lupus-like features such as splenomegaly, lymphadenopathy, and glomerulonephritis (31). These mice present with different cecal microbiota compositions depending on their age and the presence of lupus-mimicking phenotypes (12). The proportion of gut bacteria from the order *Lactobacillales* has been reported to be decreased in lupus-prone mice (12, 13). Here, the diversity and composition of gut microbiota were evaluated in mice before (6 weeks old) and after (16 weeks old) the development of a lupus-mimicking disease. The acquisition of a lupus-like phenotype was associated with significant reductions in gut bacterial diversity (**Figure 1A**), and microbial compositions (data not shown) were different compared to the pre-disease state. Data from the 16s rRNA sequencing of cecal microbiota revealed that the relative abundancies of *Lactobacillaceae* and *Lactobacillus* at the family and genus level, respectively, were significantly reduced after disease onset (**Figures 1B, C** **)**.

### LA Improves Gut Dysbiosis and Decreases the Proportion of Double Negative T Cells and Renal Inflammation in Lupus-Prone Mice

**Figure 2:**
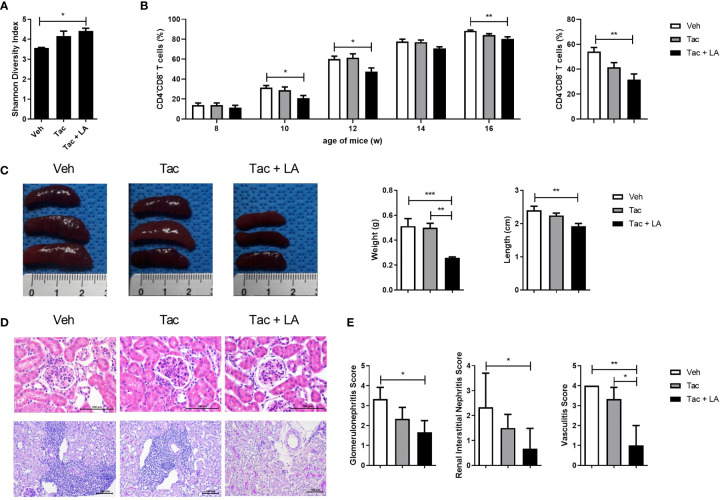
The combination of Tac and LA improves gut dysbiosis and reduces the proportion of DNT cells and kidney inflammation in lupus mice. Eight-week-old MRL/*lpr* mice (*n* = 5 in each group) were orally administered Tac (5 mg/kg, daily for 8 weeks) alone, or Tac + LA (50 mg/kg, daily for 8 weeks). **(A)** Shannon diversity indices of the cecal microbiota from MRL/*lpr* mice at 16 weeks old. **(B)** The proportion of DNT (CD4-CD8-) cells in the peripheral blood of MRL/*lpr* mice during *in vivo* experiments (left panels). The percentage of DNT cells in the spleens of 16-week-old MRL/*lpr* mice (right panels). **(C)** The weights and lengths of spleens from 16-week-old MRL/*lpr* mice. **(D)** Representative photomicrographs of haematoxylin and eosin (H&E)-stained renal tissues of 16-week-old MRL/*lpr* mice. Glomeruli and tubules (upper panels, original magnification ×400) and vascular pathologies (lower panels, original magnification ×200). **(E)** Semiquantitative inflammation scores calculated from the renal tissue sections. All experiments were performed at least three times in triplicate. Data are shown as mean ± SEM. *p < 0.05, **p < 0.01, ***p < 0.001.

Given the results presenting decreased proportion of *Lactobacillus* in lupus-prone mice and the evidence supporting *Lactobacillus* supplementation for regulating gut homeostasis and immunological imbalances in lupus according to the past studies, we considered the probiotic supplementation using one of the species from the genus *Lactobacillus* as a modality for modulating immune dysregulation and gut dysbiosis in the present study (12, 18, 19). Among various species included in the genus *Lactobacillus*, LA is suggested as one of the species exerting immune-regulatory effects in animal models for immunologic diseases such as a colitis (32). Considering such reports demonstrating the potential immune-modulatory functions of LA, this species was consequently chosen to supplement Tac treatment in the murine model for lupus. Tac (5 mg/kg) with or without LA (50 mg/kg) was administered daily to 8-week-old MRL/*lpr* mice for 8 weeks. When given alone, Tac did not sufficiently restore the reduced diversity index of cecal microbiota in the 16-week-old lupus-prone mice. By contrast, in mice treated with LA and Tac, the Shannon diversity indices were significantly improved, indicating enrichment of the cecal bacterial composition (**Figure 2A**).

SLE patients show increases in the population of cluster of CD4-CD8- T cells, also known as double negative T (DNT) (33). DNT cells produce inflammatory cytokines and infiltrate target tissues in lupus patients (34). Similar to humans, MRL/*lpr* mice also show an increase, of up to 80%, in the proportion of DNT cells (35). Notably, the proportion of DNT cells correlates with the severity of lupus-like phenotypes in MRL/*lpr* mice. Thus, changes in DNT cell proportions were assayed biweekly for the duration of the *in vivo* experiment, and the effect of the different treatments on their proportions was investigated. The percentage of DNT cells in the peripheral blood of MRL/*lpr* mice increased to over 80%. Tac-alone treatment did not significantly change the percentage of DNT cells, but when combined with LA, the proportion of DNT cells were significantly reduced (**Figure 2B**, left panels). The combination treatment also decreased the DNT cell population in splenocytes acquired from 16-week-old MRL/*lpr* mice (**Figure 2B**, right panels). Moreover, the sizes and weights of spleens, which represent the severity of the lupus-like phenotype, in MRL/*lpr* mice were significantly lower in the combination-treated mice (**Figure 2C**). These findings suggest that, in the murine model, oral administration of LA in addition to Tac could improve cecal microbial composition and alleviate lupus-like features by reducing the proportion of DNT cells.

Histopathological assessments of kidney tissue acquired from 16-week-old MRL/*lpr* mice, with and without treatment, were performed to determine whether the effects of oral probiotic administration on DNT cells also apply to renal tissue inflammation. Although Tac is prescribed to patients with membranous LN, the Tac-alone treatment did not significantly reduce renal inflammation in the lupus-prone mice. By contrast, as with systemic autoimmunity, supplementation of LA with Tac significantly reduced inflammation of murine kidneys, as reflected in the representative images for renal pathology (**Figure 2D**) and semiquantitative scores for glomerulonephritis, interstitial nephritis, and vasculitis (**Figure 2E**). These findings imply that the addition of oral probiotics augments the effects of Tac on kidney inflammation in lupus-prone mice.

### Tac and LA Combination Treatment Improves Systemic Autoimmunity in Lupus-Prone Mice

**Figure 3:**
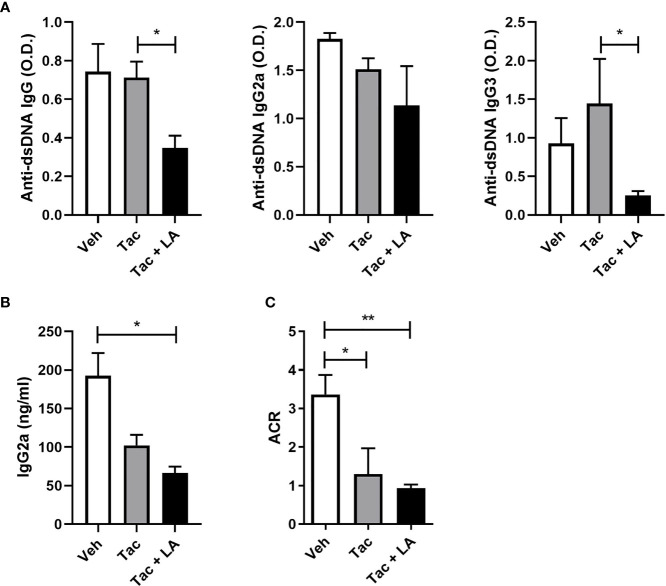
The combination of Tac and LA inhibits the production of autoantibodies in lupus-prone mice. After the *in vivo* experiments, as described in **Figure 2**, serum and urine samples were acquired from 16-week-old MRL/*lpr* mice (n = 5). **(A)** Levels of anti-dsDNA antibodies (total IgG) and their isotypes (IgG2a and IgG3) in the sera of lupus-prone mice treated with vehicle or Tac ± LA. **(B)** Levels of IgG2a in the sera from MRL/*lpr* mice. **(C)** Albumin/creatinine ratio (ACR) measured from urine samples of MRL/*lpr* mice at the age of 16 weeks. All experiments were performed at least three times in triplicate. Data are shown as mean ± SEM. *p < 0.05, **p < 0.01.

The impact of the combination treatment on systemic autoimmunity was assayed by measuring levels of IgG2a and anti-dsDNA antibodies, which are the most pathognomonic autoantibodies in lupus, as well as their isotypes in the sera of MRL/*lpr* mice (36, 37). Serum levels of total anti-dsDNA antibodies and their isotypes, IgG2a and IgG3 were measured at 16-week-old MRL/*lpr* mice. Although Tac-only treatment did not reduce their levels, it was suppressed by the combination-treatment (**Figure 3A**). Also, sera IgG2a levels were inhibited by the addition of LA (**Figure 3B**).

The immune complexes causing renal inflammation in lupus are mainly autoantibodies and immunoglobulins (38, 39). The relationship between levels of circulating autoantibodies and immunoglobulins and the severity of proteinuria was assessed. Proteinuria severity in 16-week-old MRL/*lpr* mice, as indexed by the albumin/creatinine ratio, was ameliorated by both treatments (**Figure 3C**). Accordingly, the Tac and LA combination treatment may reduce circulating autoantibodies and the production of pathogenic immunoglobulins, thereby ameliorating the renal tissue damage caused by immune complex deposition.

### Th17/Treg Balance Was Modulated by LA Supplementation

**Figure 4:**
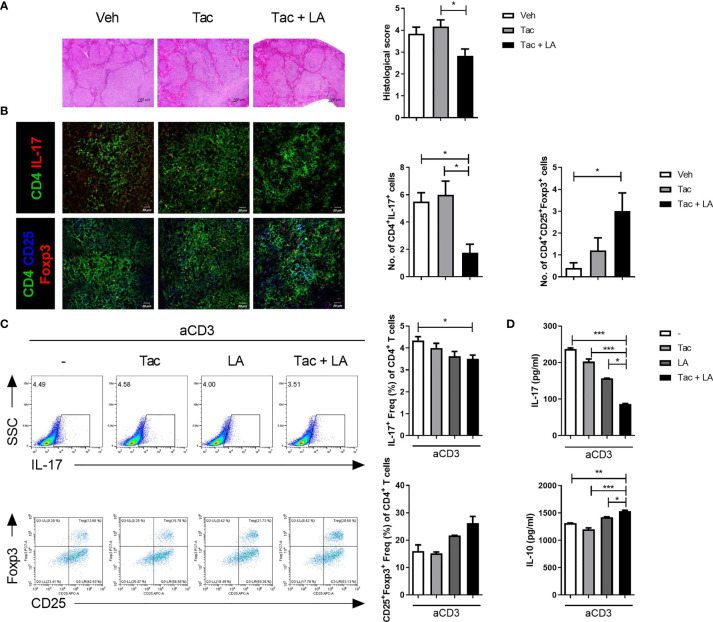
The imbalance between Th17 and Treg cells in lupus-prone mice was alleviated by the combination treatment of Tac and LA. After the *in vivo* experiments, as described in **Figure 2**, cryosectioned spleen tissues were acquired at the time of sacrifice. **(A)** Representative photomicrographs of spleen tissues stained with H&E (left panels, original magnification ×40) and their histological scores (right panels). **(B)** Confocal photomicrographs of CD4+IL-17+ cells (Th17), and CD4+CD25+Foxp3+ cells (Treg) in spleen tissues (left panels, original magnification ×200). Cell counts are shown in the right panels. **(C)** Total splenocytes extracted from the spleens of MRL/*lpr* mice were cultured with Tac (0.1 nM) and/or LA (100 μg/ml), under stimulation with anti-CD3 antibodies (0.5 μg/ml) for 96 h. Flow cytometry data of the proportion of IL-17-(upper panels) and Foxp3-expressing (lower panels) cells, expressed as the dot plots (left panels) and bar graphs (right panels). **(D)** Levels of IL-17 and IL-10 in the supernatants of splenocytes cultured under the same conditions as **(C)**. All experiments were performed at least three times in triplicate. Data are shown as mean ± SEM. *p < 0.05, **p < 0.01, ***p < 0.001.

SLE presents with an imbalance between Th17 and Treg cell populations, characterized by increased Th17 and decreased Treg cells. Despite its wide usage in lupus patients, Tac can negatively affect Treg cell differentiation (6, 8, 9). Because previous studies have reported that LA administration can modulate T cell subset balance by increasing Treg cells (19, 22), the therapeutic effects of LA when added to Tac may result from a modulated Th17/Treg ratio. Changes in the proportion of Th17 and Treg cells following Tac treatment, with and without LA supplementation, were evaluated *ex vivo* using cryosectioned spleen tissues from 16-week-old MRL/*lpr* mice treated as described above, and *in vitro* using total splenocytes from the same mice. Similar to the kidney, the spleen tissues from mice treated with the combination regimen show significantly lower inflammation (**Figure 4A**). Confocal imaging of spleen tissue from the 16-week-old mice show reduced numbers of IL-17-expressing T cells and increased numbers of Foxp3-expressing T cells in the Tac + LA-treated mice only (**Figure 4B**). In total splenocytes from lupus-prone mice that were stimulated *in vitro* with anti-CD3 antibodies and treated with Tac, LA, or Tac + LA, only the combination treatment significantly inhibited Th17 cell differentiation and showed a tendency to induce Treg cells, when compared to controls (**Figure 4C**). Notably, cells treated with Tac alone showed slightly decreased Treg differentiation relative to the control. Cytokine quantification in the supernatants of the splenocytes obtained under the aforementioned conditions revealed that the levels of inflammatory IL-17 and regulatory IL-10 were significantly decreased and increased, respectively, in the Tac + LA treated cells compared to controls, and to the Tac- and LA-alone treated cells (**Figure 4D**).

### SIGNR3 Receptor Mediates the Immune Regulatory Properties of LA

Gut microbiota express surface proteins that interact with specific receptors on the host’s immune cells, potentially affecting the systemic immune system (40). Surface layer protein (Slp) A is a unique protein expressed on the surface of LA (41). It binds to the receptor of immune cells called SIGNR3 (20). The interaction between SlpA and SIGNR3 can skew T cell differentiation towards immune regulation, leading to an increased proportion of Treg cells (20).

**Figure 5:**
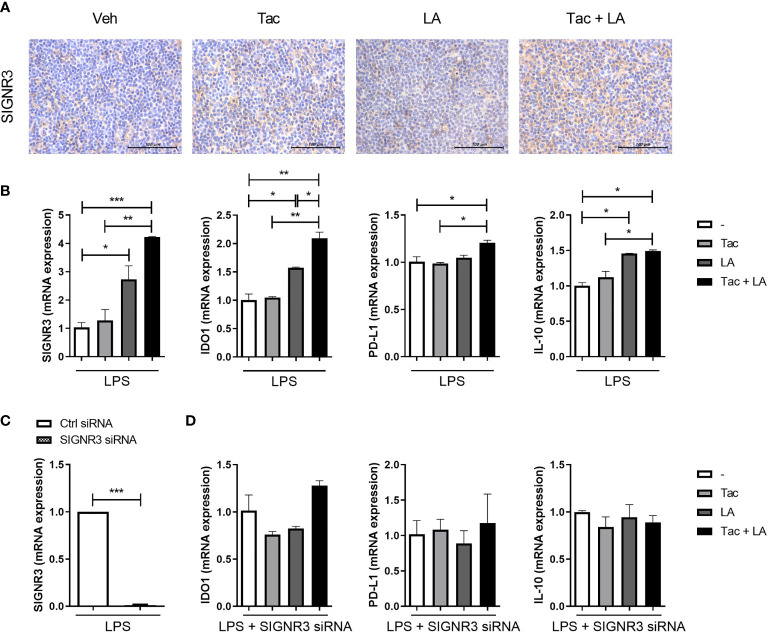
SIGNR3 receptor mediates immune regulation in lupus-prone mice treated with the combination of Tac and LA. **(A)** Representative pictures of immunohistochemical staining of SIGNR3 in spleen tissues from 16-week-old MRL/*lpr* mice treated with the same *in vivo* experiments shown in **Figure 2** (original magnification ×400). **(B)** Non-T cells from spleens of the mice treated with Tac (0.1 nM) and/or LA (100 μg/ml), were cultured and stimulated with LPS for 24 h. Then, mRNA levels of SIGNR3, IDO1, PD-L1, and IL-10 were determined using real-time PCR (qPCR). **(C)** Non-T cells from spleens of the mice were transfected with SIGNR3 siRNA after pre-treatment with LPS for 24 h. Expression levels of SIGNR3 mRNA in SIGNR3 siRNA-transfected cells. **(D)** The siRNA-transfected cells were stimulated with LPS and treated with Tac and/or LA for 24 h. Expression levels of IDO, PD-L1, and IL-10 mRNA, measured by qPCR. All experiments were performed at least three times in triplicate. Data are shown as mean ± SEM. *p < 0.05, **p < 0.01, ***p < 0.001.

To investigate the role of SIGNR3 in host-microbial interactions, the effect of probiotics on SIGNR3 expression in lupus-prone mice was first evaluated. Spleen tissues extracted from LA and Tac-treated MRL/*lpr* mice contained more SIGNR3-immunopositive cells than those from mice in the other treatment groups (**Figure 5A**). In subsequent *in vitro* experiments, LPS-stimulated splenocytes from MRL/*lpr* mice were cultured under Tac and/or LA. The splenocytes treated with LA alone or Tac + LA showed significantly higher mRNA expression levels of SIGNR3 and other immune-regulatory factors, including IDO1, PD-L1, and IL-10 (**Figure 5B**). The combination treatment with Tac and LA showed more remarkable induction of these factors than the LA alone treatment, indicating the additive effects of the combination. Next, SIGNR3 was silenced in splenocytes using siRNA transfection (**Figure 5C**). The siRNA-transfected cells did not show increased mRNA expression of regulatory cytokines, even with the combination treatment (**Figure 5D**). These results demonstrate that the immune regulatory effects of LA could be due to SIGNR3-mediated host-microbial interactions.

### The Efficacy of LA for Modulating Th17/Treg Proportions in Human Cells

**Figure 6:**
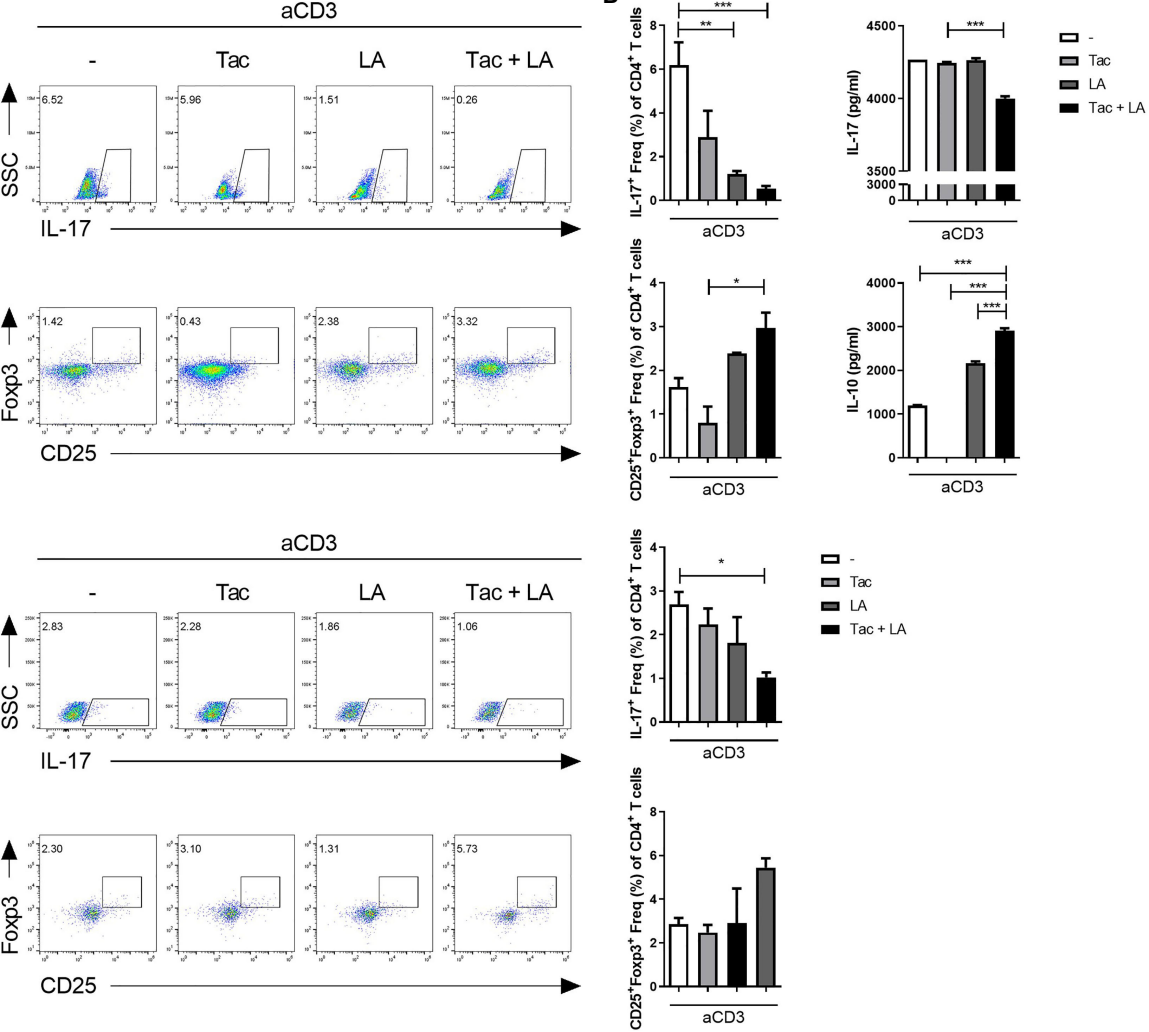
*In vitro* treatment with the combination of Tac and LA modulates the Th17/Treg balance in human peripheral blood. Peripheral blood mononuclear cells (PBMCs) were isolated from healthy humans (n = 5). Cells were stimulated with anti-CD3 antibodies and treated with Tac and/or LA for 72 h. **(A)** Flow cytometry analysis of IL-17-expressing cells (Th17 cells) and Foxp3-expressing cells (Treg cells). **(B)** Levels of IL-17 (upper panels) and IL-10 (lower panels) in the supernatants of cultured cells from the healthy humans. **(C)** PBMCs from lupus patients (n = 9) were isolated and cultured under anti-CD3 antibody stimulation and treatment with Tac and/or LA for 96 h. Flow cytometry data of the proportions of Th17 (upper panels) and Treg (lower panels) cells. All experiments were performed at least three times in triplicate. Data are shown as mean ± SEM. *p < 0.05, **p < 0.01, ***p < 0.001.

Lastly, the immune regulatory effects of LA were tested *in vitro* in human cells. Isolated PBMCs from healthy humans were stimulated with anti-CD3 antibodies in the presence of Tac, LA, or Tac + LA. After 96 h of cell culture, the Tac + LA-treated cells showed the largest reduction and elevation in IL-17+ CD4 and CD25+Foxp3+ CD4 T cell numbers, respectively (**Figure 6A**). Similar to the murine splenocytes, the Tac-alone-treated cells showed a lower proportion of Treg cells compared to the control. ELISA of the supernatants of the cells revealed that those treated with the combination of LA and Tac had the greatest decrease and increase in IL-17 and IL-10 levels, respectively (**Figure 6B**). PBMCs from SLE patients displayed analogous results under the same conditions (**Figure 6C**). These findings show the potential immunomodulatory effects of adding LA to Tac for the treatment of SLE patients.

## Discussion

In this study, probiotic supplementation of an immunosuppressant increased its efficacy in a lupus animal model by improving gut dysbiosis and modulating Th17/Treg proportions. More specifically, LA supplementation restored the cecal microbial composition in lupus-prone mice. This probiotic significantly improved the efficacy of Tac, in terms of regional inflammation and systemic autoimmunity, in lupus-mimicking mice, where the proportion of DNT cells and levels of serum autoantibodies were reduced, and renal histology was improved. Based on the present results, LA appears to modulate Th17/Treg balance by reducing proportions of Th17 cells and increasing those of Treg cells, since silencing SIGRN3 blocks its regulatory effects. This report is the first to demonstrate the potential efficacy of a lupus treatment that combines probiotic supplementation with a general immunosuppressant.

Since its efficacy was first demonstrated in murine models (42), Tac has been suggested as a treatment option for lupus, and especially for membranous LN, in the guidelines of European countries (3). However, the exact mechanisms underlying the clinical efficacy of Tac in lupus patients remain unclear. Tac is thought to globally inhibit T cell proliferation and differentiation (4, 5). IL-2, which is transcriptionally suppressed by Tac, is essential for Treg cell differentiation (43, 44), and pharmacological suppression thereof can cause an imbalance in the Th17/Treg ratio, leading to immune dysregulation (45). The immune-dysregulating effects of Tac, exerted *via* the inhibition of Treg cells, have been described in studies on organ transplantation, for which Tac is more widely used than in lupus patients. Organ transplant recipients treated with Tac were more likely to experience acute rejection, possibly due to a Tac-induced reduction in Treg cells stemming from increased Treg apoptosis and attenuation of activity in IL-2-related pathways (46). According to *in vitro* data, IL-2 supplementation could promote organ transplant survival (47). In this study, Tac inhibited Treg cell differentiation and regulatory cytokine secretion in a murine lupus model, both *in vitro* and *in vivo*. Tac-induced immune suppression, likely due to its effects on Treg cell differentiation, has a modest inhibitory effect on immune effector cell function, but its overall efficacy is limited by Treg cell shortages related to IL-2. Overall, Tac did not display noteworthy immune-modulatory effects on systemic autoimmunity, as demonstrated by the DNT cell population and serological status, nor on regional inflammation, such as in the kidney, where Tac is mainly used in lupus patients.

Several attempts have been made to compensate for Tac-related immune-dysregulation, including IL-2 replacement (47), which can lead to flare-ups of lupus-like symptoms, and inhibition of intracellular signals (48). However, the efficacy of these approaches have not been demonstrated sufficiently in humans. The immune-regulatory properties of probiotic supplementation have been widely reported in animal models of various diseases (19). Given the ease of administration in humans and lupus patients, probiotics are used as immune modulators. Species from the *Lactobacillus* family were considered as candidates for promoting Treg cell expression, based on gut microbiome profiles in lupus-prone murine models (11–13). While some *Lactobacillus* species could induce Treg cell expression (18–20, 32), other strains, such as *L. reuteri*, have been suggested to confer a protective effect in murine models, but also act as potent mediators in lupus patients (49). Therefore, the choice of bacteria should be carefully considered at the species level. Ultimately, LA, a probiotic consistently reported to have Treg-inducing effects in animal models, was used as a complementary factor for Tac (20, 22, 32). Peterson et al. previously reported that LA could promote Treg activity in a mouse model of colitis (32). The immune-regulatory effects of LA are not limited to simply enhancing Treg cell functional activity, but also include affecting the Th17/Treg balance by increasing the number of Treg cells. In this study, an immune-modulatory effect of LA was observed *in vitro*, and the *in vivo* results showed that it led to a systemic reduction in autoimmunity.

Trillions of microbiota reside within the gut mucosa and interact with the host (40). After colonization, supplemented probiotics exert their regional and systemic effects in the same manner. LA possesses three types of Slps: SlpA, SlpB, and SlpX (41). These mainly interact with pattern recognition receptors on host cells, such as dendritic cells (DCs) and macrophages. Lightfoot et al. reported that SlpA is associated with immune regulation; it binds to SIGNR3 (20), which resembles the human DC-specific ICAM-3-grabbing non-integrin (DC-SIGN) (50). Whereas the human DC-SIGN is mainly involved in allergic reactions and fungal immunity (51, 52), SIGNR3, a murine homolog, confers its regulatory properties by increasing the expression of protective cytokines, such as IL-10 (20). To confirm whether the Slps-SIGNR3 interaction is central to the immune-regulatory functions of LA, SIGNR3 was deleted in murine cells in this study, which resulted in repression of LA-mediated immune regulation. Notably, the immune-regulatory mechanisms of LA supplementation seemed to be separate from the IL-2-mediated T-cell suppression of Tac, possibly accounting for its effects on Tac efficacy. In support of this, the *in vivo* results indicated that probiotic immune regulation is independent from Tac immunosuppression, as does the gradually increasing efficacy observed in the *in vitro* experiments. However, whether *Lactobacillus* species are reduced in the gut microbiota of lupus patients according to the disease state, and whether supplementation with other bacteria possessing the same surface protein (i.e., SlpA) exert the same immune-regulatory effects in lupus, needs further study. Also, whether human DC-SIGN, a human homolog of SIGNR3, is the same target receptor of LA in lupus patients remains to be determined.

The high heterogeneity of lupus manifestations precludes the use of general immunosuppressants as a treatment. Although disease phenotype clustering and the application of specific treatments are increasing, numerous challenges remain. Given the importance of Th17/Treg imbalance in lupus pathogenesis, suppression of Treg cells by Tac, a currently available treatment for lupus, limits its therapeutic efficacy and application. This study attempted to compensate for Tac-induced Treg depletion *via* probiotic supplementation. In a murine lupus model, oral administration of LA effectively ameliorated gut dysbiosis and improved Tac efficacy by modulating the Th17/Treg ratio. In future trials, combination treatment with immunosuppressants and probiotics should be considered as a potential therapeutic modality for lupus.

## Data Availability Statement

The raw data supporting the conclusions of this article will be made available by the authors, without undue reservation.

## Ethics Statement

All procedures were approved by the ethics committee of Seoul St. Mary’s Hospital (Seoul, Korea). The patients/participants provided their written informed consent to participate in this study. The animal study was reviewed and approved by the Animal Research Ethics Committee of the Catholic University of Korea (approval number: 2020-0151-04).

## Author Contributions

DK, S-HP, M-LC, and S-KK conceived and designed the experiments. DK conducted the experiments. J-WC performed the histochemical study. DK, YP, S-HP, M-LC, and S-KK analyzed the data. DK, YP, M-LC, and S-KK wrote and revised the manuscript. All authors contributed to the article and approved the submitted version.

## Funding

This research was supported by a grant of the Korea Health Technology R&D Project through the Korea Health Industry Development Institute (KHIDI), funded by the Ministry of Health & Welfare, Republic of Korea (grant number HI20C1496) and the Bio & Medical Technology Development Program of the National Research Foundation (NRF) & funded by the Korean government (MSIT) (No. NRF-2017M3A9F3041045). Additional funding was supported by Basic Science Research Program through the National Research Foundation of Korea(NRF) funded by the Ministry of Science, ICT and future Planning (grant NRF-NRF-2018R1A2A2A05018848).

## Conflict of Interest

The authors declare that the research was conducted in the absence of any commercial or financial relationships that could be construed as a potential conflict of interest.

## Publisher’s Note

All claims expressed in this article are solely those of the authors and do not necessarily represent those of their affiliated organizations, or those of the publisher, the editors and the reviewers. Any product that may be evaluated in this article, or claim that may be made by its manufacturer, is not guaranteed or endorsed by the publisher.
